# Preclinical mouse models for *BRCA1*-associated breast cancer

**DOI:** 10.1038/sj.bjc.6605350

**Published:** 2009-09-29

**Authors:** R M Drost, J Jonkers

**Affiliations:** 1 Division of Molecular Biology, The Netherlands Cancer Institute, Plesmanlaan 121, Amsterdam 1066 CX, The Netherlands

**Keywords:** *BRCA1*, breast cancer, mouse models, tumour suppressor

## Abstract

A substantial part of all hereditary breast cancer cases is caused by *BRCA1* germline mutations. In this review, we will discuss the insights into BRCA1 functions that we obtained from mouse models with conventional and conditional mutations in *Brca1*. The most advanced models closely resemble human *BRCA1*-related breast cancer and may therefore be useful for addressing clinically relevant questions.

Breast cancer is by far the most frequent cancer in women, accounting for over 20% of all cancer cases. Familial breast cancers, including those associated with heterozygous germline mutations in the major susceptibility genes *BRCA1* and *BRCA2*, account for 5–10% of breast cancer cases in the western world. *BRCA1* mutation carriers have a lifetime risk of about 80% for developing breast cancer and a 40% lifetime risk for developing ovarian cancer. Most *BRCA1*-associated tumours show loss of heterozygosity (LOH) at the *BRCA1* locus, leading to loss of the wild-type allele, which is consistent with a tumour suppressor function of BRCA1 (Narod and Foulkes, 2004).

Since the discovery of the *BRCA1* gene in 1994 (Miki *et al*, 1994), several genetically engineered mouse models have been generated for studying the *in vivo* functions of BRCA1. Initial studies used conventional knockout mice with germline mutations in the mouse *Brca1* gene. These conventional *Brca1* mouse mutants have enabled us to learn a lot about the biological roles of BRCA1. Because of the embryonic lethality of homozygous animals carrying two defective *Brca1* alleles and the lack of mammary tumour development in heterozygous mice carrying one defective and one wild-type *Brca1* allele, these models could not be used to study the role of BRCA1 in tumorigenesis. To overcome these problems, the investigators generated conditional *Brca1* knockout mice that enable tissue-specific inactivation of BRCA1 by Cre recombinase-mediated deletion of one or more *Brca1* exons flanked by *loxP* recombination sites (Jonkers and Berns, 2002). The most recently developed conditional *Brca1* mammary tumour models closely mimic several important aspects of human *BRCA1*-associated breast cancer and therefore serve as important tools for the development of novel therapies for this disease. Before elaborating on the *Brca1* conventional and conditional mouse models that have been generated to date, we will discuss the characteristics of human *BRCA1*-related breast cancer in more detail.

## Human *BRCA1*-associated breast cancer

*BRCA1*-associated breast tumours are mostly high-grade invasive ductal carcinomas (IDCs) that lack expression of estrogen receptor (ER), progesterone receptor (PR) and ERRB2/HER2, which is referred to as ‘triple-negative’ breast cancer (Johannsson *et al*, 1997). Consequently, most patients with *BRCA1*-mutated breast cancer do not benefit from therapeutics that target ER- or ERBB2/HER2-expressing tumour cells. Gene expression profiling revealed a strong resemblance between *BRCA1*-mutated tumours and sporadic basal-type breast cancer (Sorlie *et al*, 2003). *BRCA1*-related tumours commonly express basal cytokeratins (CK5, CK6, CK14 and CK17), are highly proliferative and show pushing margins (Foulkes *et al*, 2003). *BRCA1*-mutated tumours also show a significantly higher degree of genomic instability than sporadic breast cancers (Tirkkonen *et al*, 1997), which is likely because of the functions of BRCA1 in cell-cycle regulation and DNA repair (see below).

Mutations in *BRCA1* are not confined to certain functional domains, but are scattered throughout the gene (Breast Cancer Information Core; http://research.nhgri.nih.gov/bic/). Approximately half of all *BRCA1* mutations are protein-truncating or deleterious missense mutations, whereas the pathogenic potential of the remainder is unknown (Chenevix-Trench *et al*, 2006).

Mutations in the tumour suppressor gene *TP53* are more frequent in *BRCA1*-associated breast tumours than in sporadic cases (Greenblatt *et al*, 2001), mainly because of a selective increase in protein-truncating *TP53* mutations (Holstege *et al*, 2009; Manié *et al*, 2009).

## Insights into the biological functions of BRCA1

BRCA1 has been implicated in a remarkably broad range of cellular processes and has also been reported to interact with a large number of different proteins. In this section, we will briefly describe some of the known functions of BRCA1 and also review some recent data that point towards the novel functions of BRCA1.

First, BRCA1 has been found to co-localise and interact with proteins involved in DNA repair, such as RAD51 (Scully *et al*, 1997). This interaction led to the suggestion that BRCA1 is involved in the maintenance of genomic stability through a function in DNA damage repair. The direct proof for this notion was provided by Moynahan *et al* (1999), who showed that BRCA1-deficient mouse embryonic stem (ES) cells are impaired in homology-directed repair of DNA double-strand breaks (DSBs). Further indications for a role of BRCA1 in DNA repair are the increased chromosomal instability and high sensitivity to DNA-damaging agents of BRCA1-deficient cells (Kennedy *et al*, 2004).

Besides its role in DNA repair, BRCA1 has been implicated in transcriptional regulation (through its interaction with RNA polymerase II and known transcription factors), cell-cycle progression (Deng, 2006), ubiquitination and chromatin remodelling (Mullan *et al*, 2006), as well as in maintenance of X-chromosome inactivation (Ganesan *et al*, 2002).

Recent work in the group of Wicha revealed that BRCA1 may have a role in the differentiation of ER-negative stem/progenitor cells to ER-positive luminal cells (Liu *et al*, 2008). Inhibition of BRCA1 in primary breast epithelial cells by RNA interference leads to an increase in ALDH1-positive stem/progenitor cells and a decrease in ER-positive luminal cells. Thus, loss of BRCA1 seems to induce a block in epithelial differentiation and expansion of the undifferentiated stem/progenitor cell compartment. These results might explain why most *BRCA1*-mutated breast tumours have an undifferentiated basal-like phenotype.

## Conventional *BRCA1* mouse models

A range of conventional *Brca1* knockout mouse models has been generated in an attempt to study the effects of BRCA1 loss. Until now, a total of 10 different conventional *Brca1* mouse mutants have been generated and characterised, each carrying a mutation in a different part of the gene (Xu *et al*, 1999b; Evers and Jonkers, 2006; Kim *et al*, 2006). In contrast to women with heterozygous *BRCA1* germline mutations, none of the heterozygous *Brca1* mouse mutants developed spontaneous mammary tumours. Although the reason for this inconsistency is still unclear, it could point to a species difference: the lifespan of a mouse might simply be too short or the rate of LOH might be too low for heterozygous *Brca1* mice to acquire additional mutations necessary for tumour development. Alternatively, there might be (tissue-specific) differences in haplo-insufficiency of the heterozygous *Brca1* allele between humans and mice. Embryonic lethality is observed for most homozygous *Brca1* mouse mutants. In line with the embryonic lethality of *Brca1* mouse mutants, no homozygous *BRCA1* mutation carriers have been described (Kuschel *et al*, 2001).

Most homozygous *Brca1* mouse mutants die at mid-gestation, between embryonic day 7.5 and 13.5, due to reduced cellular proliferation without signs of increased apoptosis (Evers and Jonkers, 2006). The variation in time point and penetrance of embryonic lethality could be a consequence of different genetic backgrounds of various *Brca1* mouse strains. However, the differences in protein truncation and alternative splicing of *Brca1* could also have an important role in the observed phenotypic variation between these models. A comprehensive characterisation of the regulation and function of alternative splice variants is necessary for accurate interpretation of the different *Brca1* mutant phenotypes. Evolutionary conservation may be a good indication for the functionality of specific splice variants. Thus far, three *Brca1* splice variants have been shown and functionally analysed in mice: *Brca1-*Δ*11* (Xu *et al*, 1999b; Kim *et al*, 2006), *Brca1-Iris* and *Brca1-*Δ*22* (Pettigrew *et al*, 2008).

Mouse embryos carrying *Brca1* mutations that abolish expression of full-length *Brca1* without affecting *Brca1-*Δ*11* expression survive significantly longer than embryos harbouring *Brca1* mutations that abolish expression of both transcripts (Evers and Jonkers, 2006). Mouse BRCA1-Δ11, similar to full-length BRCA1, is localised in nuclear foci and shows a cell-cycle-regulated expression pattern (Huber *et al*, 2001). However, BRCA1-Δ11 is not phosphorylated and does not promote formation of RAD51 foci upon DNA damage. Indeed, homozygous *Brca1*^*Tr*^ mouse mutants that express BRCA1-Δ11 are viable on a BALB/c genetic background, but develop various tumours including mammary carcinomas after long latency (Ludwig *et al*, 2001). Similarly, mice with mammary gland-specific deletion of full-length *Brca1* but retention of *Brca1-*Δ*11* develop mammary adenocarcinomas characterised by genetic instability (Xu *et al*, 1999a). Thus, BRCA1-Δ11 may compensate for some of the functions of full-length BRCA1 during embryogenesis, but is unable to fully execute the functions of full-length BRCA1 in maintenance of genomic stability and tumour suppression.

The *BRCA1-IRIS* transcript comprises exons 1–11 and a part of intron 11, encoding for a protein with the same N-terminus as full-length BRCA1, but with a unique C-terminus (Elshamy and Livingston, 2004). BRCA1-IRIS was shown to be exclusively chromatin associated and to have a positive influence on DNA replication. Recently, *Brca1-Iris*, the mouse orthologue of human *BRCA1-IRIS*, was identified (Pettigrew *et al*, 2008). Most BRCA1 mouse models generated to date have deleted *Brca1-Iris* in addition to full-length *Brca1* (Evers and Jonkers, 2006). Interestingly, the only *Brca1* mutation that disrupts full-length *Brca1* and *Brca1-*Δ*11* transcripts but not *Brca1-Iris*, causes embryonic lethality at E10.5 (Hohenstein *et al*, 2001), suggesting that BRCA1-IRIS cannot compensate for the loss of full-length BRCA1 and BRCA1-Δ11 expression.

Pettigrew *et al* (2008) identified *BRCA1-*Δ*22* in both human and mouse cells. Skipping of exon 22 leads to a loss of the second BRCT repeat and functional analysis revealed that the BRCA1-Δ22 protein is no longer capable of transcriptional activation. In line with this, a *Brca1* truncation mutant lacking the second BRCT repeat shows a delayed embryonic lethality when compared with *Brca1*-null mutants (Hohenstein *et al*, 2001).

Similar to the differences in time point and penetrance of embryonic lethality observed for different *Brca1* mouse mutants, the rescue of embryonic lethality by loss of p53 was also subject to phenotypic variation. In *Brca1*-null mutants, p53 deficiency resulted in only a partial rescue of embryonic lethality (Hakem *et al*, 1997; Ludwig *et al*, 1997). In hypomorphic *Brca1* mutants, the effects of a *Trp53*-null or *Trp53*-heterozygous background were more pronounced, leading to a survival of *Brca1* and *Trp53* compound mutant mice to adulthood (Cressman *et al*, 1999; Xu *et al*, 2001).

In conclusion, several *Brca1* conventional mouse mutants have been generated that show phenotypic variation, ranging from early embryonic lethality to viable mice that develop tumours. This phenotypic variation is likely due to the differences in expression of BRCA1 splice variants and BRCA1-IRIS in the various *Brca1* mouse mutants.

## Conditional *BRCA1* mouse models

Although conventional *Brca1* mouse models have enabled us to learn a lot about the biological functions of BRCA1, the observed embryonic lethality of homozygous animals and lack of mammary tumour development in heterozygous mice made it difficult to study the role of BRCA1 in tumour suppression. For this purpose, investigators turned to conditional mouse models to study the effects of BRCA1 loss.

To date, five different conditional *Brca1* alleles have been generated (Table 1): *Brca1^F11^* (Xu *et al*, 1999a), *Brca1^F5–6^* (Mak *et al*, 2000), *Brca1^F5–13^* (Liu *et al*, 2007), *Brca1^F22–24^* (McCarthy *et al*, 2007) and *Brca1^F2^* (Shakya *et al*, 2008). Although Cre-mediated deletion completely abrogates BRCA1 function for the *Brca1^F5–6^*, the *Brca1^F5–13^* and the *Brca1^F22–24^* allele, deletion of exon 11 in the *Brca1^F11^* allele does not affect the expression of the BRCA1-Δ11 isoform.

Different tissue-specific promoters were used in combination with these conditional *Brca1* alleles to achieve Cre expression in mammary epithelium. Xu *et al* (1999a) used transgenic mice expressing Cre from the whey acidic protein (WAP) or mouse mammary tumour virus (MMTV) promoter to induce mammary-specific recombination of the *Brca1*^*F11*^ alleles. In both models, different types of mammary tumours developed with a long latency and these tumours showed genomic instability and altered *Trp53* expression. The vast majority of these tumours were negative for ER, but a large proportion overexpressed ERBB2 (Table 1). Removal of one *Trp53* allele significantly reduced mammary tumour latency (Brodie *et al*, 2001). These results proved that BRCA1 functions as a tumour suppressor and cooperates with TP53 in tumorigenesis.

More evidence for interaction of BRCA1 and TP53 in tumorigenesis was provided by our lab. We generated a conditional mouse model with K14cre-mediated deletion of both *Brca1* and *Trp53* in several epithelial tissues, including mammary epithelium (Liu *et al*, 2007). Female mice of this strain showed a high incidence of mammary carcinomas that displayed important hallmarks of human *BRCA1*-associated breast tumours: tumours were poorly differentiated, highly proliferative, genomically instable, ER-negative and showed an increased expression of basal epithelial markers (Table 1).

Another mouse model for basal-like breast cancer was generated by conditional deletion of *Brca1* exons 22–24 (which harbour the second BRCT domain) in the mammary gland by using β-lactoglobin (BLG)-cre (McCarthy *et al*, 2007). When combined with heterozygosity for a *Trp53* mutation, this led to mammary tumour formation. The resulting mammary tumours were characterised by high histological grade, central necrotic areas and expression of basal-like markers. In addition, they frequently lacked expression of ER, PR and ERBB2 (Table 1). Because of their strong resemblance to human BRCA1-related breast cancer, especially the mouse models of Liu and McCarthy should prove useful in preclinical therapeutic intervention studies.

BRCA1 also interacts with BARD1, a protein that is structurally related to BRCA1 in that it contains an N-terminal RING domain and C-terminal BRCT repeats (Wu *et al*, 1996). The BRCA1/BARD1 heterodimer functions as a ubiquitin E3 ligase that can target proteins for destruction by transferring ubiquitin to these proteins (Hashizume *et al*, 2001). Until recently, the role of the BRCA1/BARD1 heterodimer in tumour suppression had not been evaluated. To address this question, Shakya *et al* (2008) generated mouse strains carrying conditional alleles of *Bard1* and/or *Brca1* and used Cre-mediated recombination to inactivate these genes specifically in mammary epithelial cells. Breast tumours arising in these conditional *Bard1*- and/or *Brca1*-mutant mice were indistinguishable from each other. These findings indicate that BARD1 itself is a tumour suppressor and that the tumour suppressor activities of BRCA1 are mediated by the BRCA1/BARD1 heterodimer.

Recent experiments have shown that ES cells expressing a ubiquitin ligase-deficient BRCA1-I26A mutant are viable and do not undergo spontaneous chromosomal rearrangements (Reid *et al*, 2008). These cells show higher levels of genomic rearrangements after mitomycin C (MMC) treatment, but do not show hypersensitivity to MMC. *Brca1*^I26A^-mutant ES cells form RAD51 foci in response to irradiation and are capable of repairing double-strand breaks by homologous recombination. These results suggest that the function of BRCA1 in the maintenance of genomic stability is not dependent on its ubiquitin ligase activity. Mouse models carrying ubiquitin ligase-deficient *Brca1* alleles should reveal whether this activity is also dispensable for the tumour suppressor activity of BRCA1.

## Chemoprevention studies in BRCA1 models

Although genetic testing for inherited *BRCA1* mutations provides valuable information to women at high risk of breast cancer, carriers of *BRCA1* mutations have few clinical options to reduce their cancer risk. Prophylactic surgery is still one of the most important measures of breast cancer prevention for *BRCA1* mutation carriers. The rationale for antihormonal therapy as an alternative for prophylactic surgery comes from the observation that oophorectomy prevents breast cancer in *BRCA1* mutation carriers (Narod and Offit, 2005). These data indicate that, despite the fact that most *BRCA1*-mutated tumours are ER-negative, tumour development in *BRCA1* mutation carriers is hormone dependent. This hormone dependency might also be the reason why BRCA1 specifically functions as a tumour suppressor in hormone-sensitive tissues such as breast and ovaries. Although the mechanistic basis for the hormone dependency and tissue specificity of *BRCA1*-associated tumorigenesis is still unknown, BRCA1 has been shown to interact directly with ER-*α* and PR and to modulate their transcriptional activities (Fan *et al*, 1999; Katiyar *et al*, 2006). To address the role of PR signalling in BRCA1-mediated carcinogenesis, Poole *et al* (2006) made use of the *WAPcre;Brca1*^*F11/F11*^;*Trp53*^*F5–6/F5–6*^ mouse model. Treatment of 3- to 4-month-old mice with the PR inhibitor mifeprestone (RU 486) prevented mammary tumour formation in these mice. Although the results obtained with this conditional *Brca1* mouse model hold promise for the development of anti-progesterones as prophylactic therapy for *BRCA1*-associated breast cancer, the jury is still out on this for several reasons. First, mifeprestone is not a selective PR antagonist because it also binds with high affinity to glucocorticoid receptors. It is therefore possible that the prevention of mammary tumours is (in part) caused by the antiglucocorticoid effects of mifeprestone. Second, it is not clear whether the mammary tumours arising in this *WAPcre;Brca1*^*F11/F11*^*;Trp53*^*F5–6/F5–6*^ mouse model do or do not express ER, PR and ERBB2. The status of ER, PR and ERBB2 could have an important role in the effectiveness of anti-progesterone therapy. Most human *BRCA1*-mutated breast cancers are ‘triple-negative’ tumours that do not express ER, PR and ERBB2. It is unclear whether anti-progesterone therapy will also protect against development of triple-negative breast tumours in *BRCA1*-mutation carriers. It may therefore be important to evaluate the effects of PR antagonists in *Brca1* mouse models that certainly recapitulate development of triple-negative *BRCA1*-associated breast cancer.

## Chemotherapeutic interventions in BRCA1 models

Breast cancers of *BRCA1* mutation carriers frequently show poor responses to neoadjuvant therapy with docetaxel, whereas platinum-based chemotherapy seems to be highly effective (Byrski *et al*, 2008, 2009). Similarly, *BRCA1/2* mutation carriers with ovarian cancer show higher response rates and longer overall survival after platinum-based chemotherapy than nonhereditary patients (Ben David *et al*, 2002; Tan *et al*, 2008).

Unfortunately, the experiments studying drug response and especially drug resistance in human patients are very time consuming. With regard to this time issue, conditional *Brca1* mouse models that develop mammary tumours with strong resemblance to human *BRCA1*-mutated breast tumours (Liu *et al*, 2007; McCarthy *et al*, 2007) can be very helpful in predicting response and resistance to conventional and targeted therapeutics. Our *K14cre;Brca1*^*F5–13/F5–13*^*;Trp53*^*F2–10/F* 2–10^ mouse model was used for studying responses to various conventional chemotherapeutics, such as doxorubicin, docetaxel and cisplatin, and for analysing the mechanisms of acquired resistance (Rottenberg *et al*, 2007). Similar to the human situation, heterogeneity in the response of individual mouse mammary tumours was observed, but eventually all tumours became resistant to doxorubicin and docetaxel. The upregulation of ATP-binding cassette (ABC) drug transporters seemed to be the main mechanism responsible for resistance to doxorubicin. Remarkably, acquired resistance to platinum compounds was never observed. However, the tumours could also not be completely eradicated; even after dose-dense platinum therapy, the tumours appeared to regrow from a small fraction of surviving cells. Currently, these platinum-resistant tumour remnants are being further characterised. In addition, attempts are being made to achieve eradication of this small fraction of surviving cells by combination therapies.

Especially intriguing is the observation that platinum resistance is never observed in these mouse tumours, whereas resistance is a major problem in the clinic. As described earlier, BRCA1 has an important role in the error-free repair of double-stranded DNA breaks that occur after platinum therapy. These mouse tumour data raise the question whether platinum resistance can occur at all in BRCA1-deficient tumours that are completely defective in homology-directed DNA repair. This question became even more evident when Swisher *et al* (2008) showed that acquired resistance to platinum compounds in *BRCA1*-mutated human ovarian tumours is associated with secondary mutations in *BRCA1* that restore the open-reading frame in platinum-resistant tumours. Three out of five platinum-resistant tumours showed secondary genetic changes in *BRCA1*, whereas no *BRCA1* alterations were observed in three platinum-sensitive tumours. The main difference between the human situation and the *K14cre;Brca1*^*F5–13/F5–13*^*;Trp53*^*F2–10/F* 2–10^ mouse model is that the mouse tumours have a homozygous deletion of *Brca1* exons 5–13. As a result of this large deletion, secondary mutations in *Brca1* cannot restore Brca1 function and serve as a mechanism for platinum resistance in the mouse tumours. Together, the human and mouse data suggest that BRCA1 not only functions as a tumour suppressor, but is also required for development of resistance to therapy.

Intervention studies with conventional chemotherapeutics were also carried out in *WAPcre;Brca1*^*F11/F11*^*;Trp53*^*F5–6/F5–6*^ and *MMTVcre;Brca1*^*F11/F11*^*;Trp53*^*F5–6/F5–6*^ models (Shafee *et al*, 2008). In line with data obtained from the *K14cre;Brca1*^*F5–13/F5–13*^*;Trp53*^*F2–10/F* 2–10^ model, *Brca1*^Δ*11/*Δ*11*^*;Trp53*^Δ*5–6/*Δ*5–6*^ tumours also responded better to platinum compounds than to doxorubicin. Following initial regression, tumours relapsed at the same site at 2–3 months after treatment. Whereas platinum resistance was never observed in the *K14cre;Brca1*^*F5–13/F5–13*^*;Trp53*^*F2–10/F* 2–10^ model, Shafee *et al* (2008) observed platinum resistance in their *Brca1*^*F11/F11*^*;Trp53*^*F5–6/F5–6*^ models. After a second round of treatment with platinum drugs, tumours recurred with a faster growth rate. This rapid recurrence could suggest the existence of a population of platinum-resistant cells that are selected during a second round of platinum treatment. An important difference between the two studies described above is that Rottenberg *et al* (2007) used a mouse model with a conditional *Brca1* null allele, whereas Shafee *et al* (2008) used a *Brca1* hypomorphic allele that still expresses the *Brca1*-Δ*11* isoform after Cre-mediated deletion of exon 11. Furthermore, the platinum treatment regime differs considerably between the two studies. It would be informative to see whether *Brca1*^Δ*11/*Δ*11*^*;Trp53*^Δ*5–6/*Δ*5–6*^ tumours would acquire full resistance to platinum drugs during additional rounds of therapy and if so, by which mechanism.

## Interventions with targeted therapeutics in BRCA1 models

Until now, targeted therapeutics are only available for ER- and ERBB2-positive breast cancer, and no tailored therapy exists for triple-negative breast cancer. As mentioned earlier, BRCA1 deficiency causes defects in homology-directed DSB repair. A few years back, BRCA1/2-deficient cells were shown to be highly sensitive to chemical inhibitors of Poly(ADP-ribose) polymerase-1 (PARP1), a key molecule in the repair of DNA single-strand breaks (SSBs) (Farmer *et al*, 2005). It is thought that, upon inactivation of SSB repair by PARP inhibition, DSBs are induced by replication fork collapse at SSBs during S phase. Therefore, PARP inhibition may be synthetically lethal with BRCA1 loss and serve as a specific therapy for *BRCA1*-mutated tumours. The *K14cre;Brca1*^*F5–13/F5–13*^*;Trp53*^*F2–10/F2–10*^ mouse model was used to study the effects of PARP inhibition in a ‘realistic’ *in vivo* setting (Rottenberg *et al*, 2008). The BRCA1-deficient tumours arising in this model showed a prolonged response to the clinical PARP inhibitor olaparib without signs of toxicity. Eventually, long-term treatment with olaparib resulted in resistance as a consequence of upregulation of the P-glycoprotein drug efflux pump. Combining platinum therapy with PARP inhibition increased the relapse-free survival as compared with platinum monotherapy, suggesting that PARP inhibition enhances the effects of DNA-damaging agents. Recently, it was shown that olaparib has antitumour activity in patients with *BRCA1-* or *BRCA2*-associated malignancies (Fong *et al*, 2009). These findings illustrate how *Brca1* conditional mouse models can be of use for preclinical assessment of new targeted therapeutics.

## Next-generation BRCA1 models

Despite the fact that current *Brca1* mouse models have enabled us to learn a lot about BRCA1 function in normal development and tumorigenesis, improvements can still be made.

For instance, nearly all existing mouse models for *BRCA1*-associated breast cancer described earlier are based on co-mutation of *Trp53* and *Brca1*. It might be possible that mutations in yet other genes, such as *Pten* (Saal *et al*, 2008), are required to effectively model *BRCA1*-mutated breast cancer in mice.

Current *Brca1* mouse models are also not ideally suited to study mechanisms of acquired resistance to conventional and targeted therapeutics. Rottenberg *et al* (2007, 2008) showed that upregulation of drug efflux pumps is the most prevalent mechanism of acquired resistance to conventional and targeted therapies for mammary tumours arising in the *K14cre;Brca1*^*F5–13/F5–13*^*;Trp53*^*F2–10/F2–10*^ mouse model. Currently, the treatment responses in this *K14cre;Brca1*^*F/F*^*;Trp53*^*F/F*^ mouse model are being studied in a P-glycoprotein-deficient background to unravel P-glycoprotein-independent mechanisms of drug resistance (Table 2).

Mouse models based on deleterious missense or protein-truncating *Brca1* mutations that closely mimic human *BRCA1* germline mutations will be useful for studying tumorigenesis, treatment responses and acquired resistance associated with known pathogenic *BRCA1* mutations. These mouse models could, for example, be used for studying the role of genetic reversion in therapy resistance (Table 2). As the number of therapy-resistant tumour samples from patients with specific *BRCA1* founder mutations is low, the mouse models carrying these mutations could offer a larger platform to study if and how the genetic reversion occurs as a mechanism of drug resistance for different *BRCA1* founder mutations. Eventually, insights gained from mouse models carrying specific *Brca1* mutations could lead to tailored therapy for people with a particular *BRCA1* mutation.

Another option is to study the consequences of individual mutations in the human *BRCA1* gene *in vivo* by creating mice that express human BRCA1. Already in 2001 it was shown that human *BRCA1* is able to rescue embryonic lethality in *Brca1* knockout mice (Chandler *et al*, 2001). This shows that it may be possible to introduce human *BRCA1* BAC clones harbouring specific mutations in an intact animal model system. This system would provide the best possible *in vivo* analysis of the phenotypic consequences of specific *BRCA1* mutations.

## Concluding remarks

Genetically engineered mouse models for BRCA1 deficiency have proven to be of critical importance for gathering insights on the diverse biological functions of BRCA1, both in normal development and tumorigenesis. In the past years, these mouse models have been further improved to recapitulate the salient features of human *BRCA1*-associated breast cancer, such as ‘triple-negative’ status, increased genomic instability and increased expression of basal epithelial markers.

Recapitulation of these characteristics in mouse models is crucial for preclinical development of chemoprevention strategies and tailored therapies for *BRCA1*-associated breast cancer. The first studies with targeted therapeutics in validated BRCA1 models have been conducted, showing excellent initial responses but P-glycoprotein-mediated drug resistance upon prolonged treatment with the PARP inhibitor olaparib (Rottenberg *et al*, 2008). Of course, it is important to keep in mind that data obtained from mouse tumour models are not necessarily predictive for clinical responses and acquired resistance in human cancer patients. Although genetically engineered mice for BRCA1 deficiency are promising preclinical models, their predictive value remains to be determined.

The PARP inhibitor olaparib was recently evaluated in a phase I clinical trial and showed antitumour activity in *BRCA1* or *BRCA2* mutation carriers with ovarian, breast or prostate cancer (Fong *et al*, 2009). In this case, the mouse data did seem to reflect the clinical response quite accurately. It will be of great interest to see whether the acquired resistance to the PARP inhibitor that we observe in the mouse model will also arise in the human situation.

Of course, current mouse models are not perfect yet and can still be further improved to closely mimic additional aspects of human *BRCA1*-related breast cancer, to study, for example, the role of genetic reversion in therapy resistance. It can be expected that the resulting models will be of even greater use in the development of therapies directed against various aspects of the disease.

## Figures and Tables

**Table 1:** Conditional *Brca1* mouse models

	***Brca1* mutation**	**p53 co-mutation**	**Cre transgene**	**Genetic background**	**Mean tumor latency (months)**	**Triple-negative tumours**	**Basal -like tumours**	**Initial platinum sensitivity**	**Platinum resistance**	**Initial PARPi sensitivity**	**PARPi resistance**
*MMTVcre;Brca1*^*F11/*Δ*11*^* WAPcre;Brca1*^*F11/*Δ*11*^ (Xu *et al*, 1999a)	*Brca1* ^Δ*11*^	No	*MMTVcre* or *WAPcre*	NIH-BL(S)	>13	—	No^a^	—	—	—	—
*MMTVcre; Brca1*^*F11/F11;p53+/−*^* WAPcre; Brca1*^*F11/F11;p53+/−*^ (Brodie *et al*, 2001)	*Brca1* ^Δ*11*^	*p53* ^*Null*^	*MMTVcre* or *WAPcre*	NIH-BL(S), C57BL/6, 129/Sv	8	No^b^	No^a^	—	—	—	—
*WAPcre*^*c*^*;Brca1*^*F11/F11*^*; p53*^*F5–6/F5–6*^ (Poole *et al*, 2006)	*Brca1* ^Δ*11*^	*p53* ^Δ*5–6*^	*WAPcre* ^*c*^	C57BL/6, 129/Sv or C57BL/6, BALB/c	7	No^c^	No^a^	Yes	Yes	—	—
*BLGcre; Brca1*^*F22–24/F22–24*^*; p53*^*+/−*^ (McCarthy *et al*, 2007)	*Brca1* ^Δ*22–24*^	*p53* ^*Null*^	*BLGcre*	C57BL/6, 129/Sv	7	Yes	Yes	—	—	—	—
*K14cre; Brca1*^*F5–13/F5–13*^*; p53*^*F2–10/F2–10*^ (Liu *et al*, 2007)	*Brca1* ^Δ*5–13*^	*p53* ^Δ*2–10*^	*K14cre*	FVB, 129/Ola	7	Yes	Yes	Yes	No	Yes	Yes
*WAPcre;Brca1*^*F1/F1*^ (Shakya *et al*, 2008)	*Brca1* ^Δ*1*^	No	*WAPcre*	C57BL/6, 129/Sv	17	Yes	Yes	—	—	—	Yes

Abbreviations: —= not determined.

a Heterogeneous mammary tumour spectrum.

b ERBB2-positive and ER-negative, PR status not determined.

c PR-positive, ER and ERBB2 status not determined.

**Table 2:** Next-generation *Brca1* mouse models for studying drug resistance mechanisms

**Mouse model**	**Conditional *Brca1* models carrying *Brca1* truncation alleles**	**Conditional *Brca1* models carrying large *Brca1* deletions**	**Conditional *Brca1* models on a Pgp1-deficient background**
*Resistance mechanism*			
Genetic reversion	Yes	No	No
Pgp1 activation	Yes	Yes	No
Other	Yes	Yes	Yes
